# Development and validation of a predictive model for sarcopenia in patients with type 2 diabetes based on the synergistic mechanism of insulin resistance and inflammation

**DOI:** 10.3389/fphys.2026.1922889

**Published:** 2026-09-21

**Authors:** Ajinisha Akeaji, Baihetinisha Akeaji, Yihe Han, Ning Wang

**Affiliations:** 1 Department of General Internal Medicine I, The First Affiliated Hospital of Xinjiang Medical University, Urumqi, China; 2 Department of Diagnostic Ultrasound, The First People’s Hospital of Kashi, Kashgar, China

**Keywords:** type 2 diabetes mellitus, sarcopenia, machine learning, insulin resistance, inflammatory marker

## Abstract

**Background:**

Type 2 diabetes mellitus (T2DM) complicated by sarcopenia represents a considerable public health challenge, yet early detection remains challenging due to a lack of practical screening tools. The aim of this study was to develop and internally validate a clinically feasible, mechanism-based machine learning model for predicting sarcopenia risk in patients with T2DM.

**Methods:**

A total of 904 patients with T2DM treated at the First Affiliated Hospital of Xinjiang Medical University from May 2024 to May 2026 were retrospectively enrolled. Sarcopenia was diagnosed according to the Asian Working Group for Sarcopenia (AWGS) 2025 consensus. The dataset was randomly partitioned into training (70%) and internal validation (30%) sets. Key predictive features were identified by intersecting the variables selected via least absolute shrinkage and selection operator (LASSO) regression and the Boruta algorithm. Eight machine learning algorithms were subsequently developed and evaluated.

**Results:**

The prevalence of sarcopenia among the patients with T2DM was 29.20%. Eight predictors were selected: age, sex, body mass index, serum albumin, alanine aminotransferase, hemoglobin, metabolic score for insulin resistance, and monocyte-to-high-density lipoprotein cholesterol ratio. LightGBM demonstrated the best overall predictive performance in the validation set, yielding an area under the receiver operating characteristic curve (AUC) of 0.928, an accuracy of 0.882, a sensitivity of 0.897, a specificity of 0.876, a precision of 0.745, and an F1 score of 0.814. Calibration and decision curve analysis indicated that the model yielded clinical net benefit. A web-based calculator developed using LightGBM enabled three-tier risk stratification: low (<15%), moderate (15%–40%), and high (>40%).

**Conclusion:**

A machine learning model for predicting sarcopenia risk in patients with T2DM was developed and internally validated, resulting in the implementation of an online risk calculator. This mechanism-based model demonstrated promising internal predictive performance and may provide a practical and cost-effective screening approach for diabetes management.

## 1 Introduction

Type 2 diabetes mellitus (T2DM) is a major global public health issue. According to the 11th edition of the Atlas published by the International Diabetes Federation (IDF), the global prevalence of diabetes among adults was projected to reach 11.11% in 2024, affecting 589 million people (Genitsaridi et al., 2026). Sarcopenia, a condition characterized by the progressive, age-associated loss of skeletal muscle mass and strength, leads to adverse outcomes such as physical dysfunction, falls, and death (Chen et al., 2025). In Asian populations, the prevalence of T2DM complicated by sarcopenia is as high as 23% (Yogesh et al., 2025), and individuals with diabetes have an approximately 1.5-fold higher risk of developing sarcopenia than those without diabetes (Chung et al., 2021).

T2DM and sarcopenia share interrelated pathophysiological mechanisms (Tack et al., 2024; Bai et al., 2025), including insulin resistance, chronic low-grade inflammation, mitochondrial dysfunction, and oxidative stress, all of which promote the loss of skeletal muscle mass and function. Among these, insulin resistance and chronic inflammation form a bidirectional positive feedback loop through a “metabolic–inflammatory coupling” mechanism, jointly driving the progression of sarcopenia in patients with T2DM (Ghosh et al., 2026).

Although the 2025 Asian Working Group for Sarcopenia (AWGS) consensus introduced a lower diagnostic age threshold of 50 years (Chen et al., 2025), early identification of sarcopenia in patients with T2DM remains challenging in clinical practice. Given the multifactorial nature of T2DM associated sarcopenia and potential nonlinear interactions among variables, machine learning may offer advantages in integrating multidimensional clinical data and identifying complex predictive patterns. Machine learning methods have demonstrated superior performance in predicting diabetic complications, including diabetic nephropathy, and diabetic foot outcomes (Fang et al., 2025; Liu et al., 2025a; Yan et al., 2025). However, although predictive models have been developed in this field, most rely on traditional regression methods and do not incorporate comorbidity-related mechanisms (Zou and Shao, 2024).

Therefore, a machine learning prediction model for sarcopenia in patients with T2DM was developed and internally validated using real-world data in accordance with the latest diagnostic consensus. Eight supervised learning models were constructed using clinically accessible surrogate markers of insulin resistance and composite inflammation indices. The aim of this study was to provide clinicians with a feasible, mechanism-based, interpretable tool along with its accompanying online risk calculator, thus contributing novel strategies for the early identification and comprehensive management of comorbid sarcopenia.

## 2 Materials and methods

### 2.1 Study population

A total of 904 patients with T2DM treated at the First Affiliated Hospital of Xinjiang Medical University between May 2024 and May 2026 were retrospectively enrolled. The inclusion criteria were: (1) age ≥50 years; (2) completion of bioelectrical impedance analysis (BIA) for skeletal muscle mass measurement and grip strength testing to evaluate sarcopenia status; and (3) availability of complete clinical data. The exclusion criteria were: (1) presence of concurrent diseases that could induce secondary sarcopenia; (2) acute conditions, such as acute infections or acute cardiovascular events; (3) missing key clinical data; and (4) use of medications affecting muscle metabolism within the preceding 3 months. The study protocol was approved by the Institutional Review Board of the First Affiliated Hospital of Xinjiang Medical University (approval No. K202509-51). Written informed consent was not required from the participants in accordance with the local legislation and institutional requirements (**Figure 1**).

**Figure 1:**
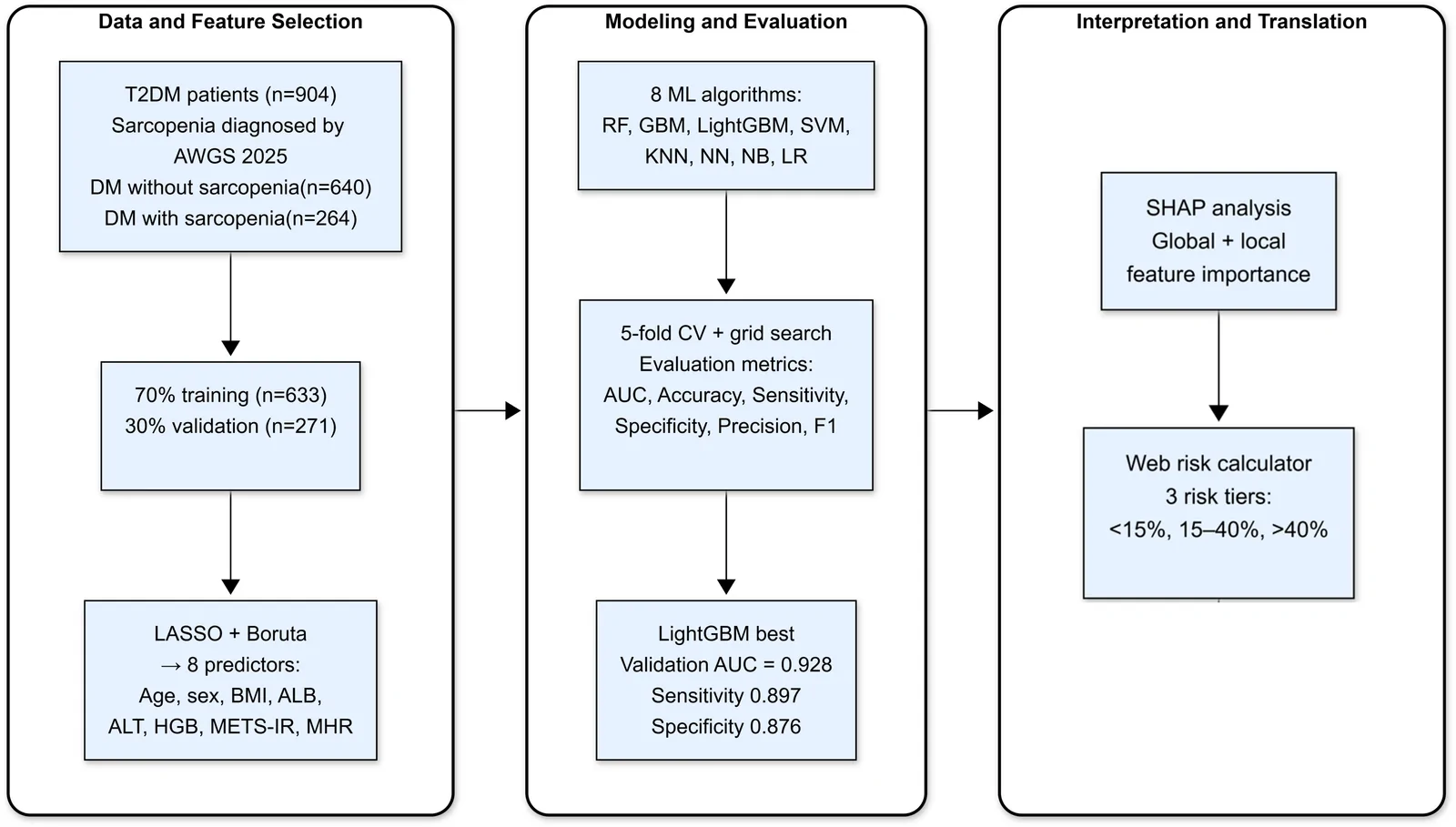
Complete analysis flow chart.

### 2.2 Data collection

Demographic characteristics, lifestyle factors, hypertension status, anthropometric measurements, and laboratory parameters (detailed in **Table 1**) were extracted from the electronic medical record system of the hospital. Surrogate markers of insulin resistance and composite inflammation indices were subsequently calculated, including the triglyceride–glucose (TyG) index, the metabolic score for insulin resistance (METS-IR), the TyG–body mass index (TyG-BMI) composite, the triglyceride-to-high-density lipoprotein cholesterol ratio (TG/HDL-C), the monocyte-to-high-density lipoprotein cholesterol ratio (MHR), the neutrophil-to-lymphocyte ratio (NLR), the systemic immune-inflammatory index (SII), and the systemic inflammatory response index (SIRI). The definitions and corresponding formulas for these indicators are provided in **Supplementary Table S1**.

**Table 1:** Baseline characteristics of the study participants.

Variable	Total (N = 904)	DM without sarcopenia (n = 640)	DM with sarcopenia (n = 264)	P	Training (n = 633)	Validation (n = 271)	P
Gender, n (%)				<0.001			0.250
Female	305 (33.7)	243 (38.0)	62 (23.5)	–	206 (32.5)	99 (36.5)	–
Male	599 (66.3)	397 (62.0)	202 (76.5)	–	427 (67.5)	172 (63.5)	–
Exercise frequency, n (%)				0.209			0.270
≥3 times/week	380 (42.0)	278 (43.4)	102 (38.6)	–	274 (43.3)	106 (39.1)	–
≤3 times/week	524 (58.0)	362 (56.6)	162 (61.4)	–	359 (56.7)	165 (60.9)	–
Hypertension, n (%)				0.305			0.203
No	564 (62.4)	392 (61.2)	172 (65.2)	–	386 (61.0)	178 (65.7)	–
Yes	340 (37.6)	248 (38.8)	92 (34.8)	–	247 (39.0)	93 (34.3)	–
Smoking, n (%)				0.023			0.627
No	652 (72.1)	476 (74.4)	176 (66.7)	–	453 (71.6)	199 (73.4)	–
Yes	252 (27.9)	164 (25.6)	88 (33.3)	–	180 (28.4)	72 (26.6)	–
Alcohol consumption, n (%)				0.046			0.585
No	617 (68.3)	450 (70.3)	167 (63.3)	–	428 (67.6)	189 (69.7)	–
Yes	287 (31.7)	190 (29.7)	97 (36.7)	–	205 (32.4)	82 (30.3)	–
Age (years)	63.00(56.75–74.00)	66.00 (58.00–75.00)	60.00 (55.00–68.00)	<0.001	64.00 (57.00–74.00)	63.00 (56.00–74.00)	0.891
BMI (kg/m²)	25.55 (23.4–27.9)	26.50(23.87–28.7)	24.20 (22.3–25.72)	<0.001	25.50 (23.40–27.70)	25.70 (23.30–28.20)	0.366
Waist circumference (cm)	92.00 (85.00–98.00)	93.00 (86.00–100.00)	90.00 (85.00–95.00)	<0.001	92.00 (86.00–97.60)	92.00 (85.00–100.00)	0.890
Hip circumference (cm)	100.00 (95.00–105.00)	100.00 (95.00–105.25)	97.50 (92.00–103.00)	<0.001	100.00 (95.00–105.00)	100.00 (94.00–105.00)	0.746
Waist–to–hip ratio	0.91 (0.88–0.96)	0.92 (0.88–0.97)	0.90 (0.89–0.94)	0.048	0.91 (0.88–0.96)	0.91 (0.88–0.97)	0.917
HbA1c (%)	7.00 (6.33–8.25)	6.98 (6.30–8.10)	7.09 (6.41–8.37)	0.222	7.03 (6.39–8.27)	6.90 (6.25–8.18)	0.351
Total cholesterol (mmol/L)	4.29 (3.50–5.03)	4.30 (3.49–5.09)	4.22 (3.50–4.87)	0.272	4.25 (3.47–4.93)	4.43 (3.59–5.18)	0.019
LDL–C (mmol/L)	2.71 (2.09–3.29)	2.68 (2.09–3.29)	2.75 (2.13–3.29)	0.309	2.68 (2.05–3.27)	2.80 (2.14–3.35)	0.084
ALB (g/L)	42.70 (39.70–45.10)	43.88 (40.70–46.00)	40.50 (37.60–42.82)	<0.001	42.70 (39.70–45.00)	42.62 (39.65–45.35)	0.808
ALT (U/L)	21.50 (16.16–29.66)	21.22 (15.80–29.31)	22.50 (17.17–31.32)	0.104	21.96 (16.80–28.80)	21.24 (15.20–31.50)	0.656
AST (U/L)	19.92 (16.80–24.05)	19.80 (16.70–24.13)	20.16 (17.39–23.82)	0.698	19.80 (17.00–24.00)	20.40 (16.65–24.12)	0.968
Uric acid (μmol/L)	311.80 (264.75–372.00)	314.00 (271.78–380.12)	301.92 (255.60–357.72)	0.014	309.00 (264.00–372.00)	317.00 (269.00–370.92)	0.274
Urea (mmol/L)	5.70 (4.70–6.90)	5.58 (4.60–6.77)	5.95 (4.90–7.12)	0.004	5.68 (4.70–6.80)	5.70 (4.80–7.05)	0.368
Creatinine (μmol/L)	65.53 (56.00–77.00)	65.10 (56.00–78.00)	66.00 (57.00–74.16)	0.909	65.80 (57.00–77.20)	64.20 (55.20–75.35)	0.256
eGFR (mL/min/1.73 m²)	95.09 (88.05–101.56)	94.69 (88.09–102.06)	95.60 (87.90–100.89)	0.726	94.80 (87.70–101.27)	95.80 (88.97–102.39)	0.138
HGB (g/L)	145.00 (131.00–155.00)	146.50 (138.00–156.00)	132.00 (115.00–152.00)	<0.001	145.00 (132.00–156.00)	145.00 (129.00–155.00)	0.558
25-hydroxyvitamin D3 (nmol/L)	50.50 (35.69–63.34)	52.31 (35.80–64.98)	45.88 (35.24–58.49)	0.017	50.42 (36.26–62.80)	50.60 (33.20–64.19)	0.786
TyG index	9.00 (8.54–9.52)	8.96 (8.51–9.48)	9.12 (8.61–9.64)	0.026	8.99 (8.56–9.48)	9.03 (8.47–9.56)	0.929
METS-IR	2.49 (2.33–2.66)	2.47 (2.32–2.65)	2.54 (2.38–2.70)	<0.001	2.50 (2.34–2.66)	2.47 (2.31–2.65)	0.125
TyG-BMI	231.56 (206.47–258.52)	237.37 (209.75–268.09)	219.84 (199.04–240.85)	<0.001	231.28 (206.82–255.76)	231.81 (205.43–267.48)	0.364
TG/HDL-C	1.50 (0.95–2.35)	1.39 (0.91–2.25)	1.69 (1.09–2.68)	<0.001	1.50 (0.99–2.32)	1.44 (0.88–2.52)	0.659
MHR	0.45 (0.34–0.62)	0.42 (0.32–0.57)	0.56 (0.39–0.69)	<0.001	0.45 (0.34–0.62)	0.45 (0.33–0.61)	0.434
NLR	1.82 (1.42–2.34)	1.83 (1.43–2.33)	1.82 (1.38–2.41)	0.443	1.83 (1.43–2.35)	1.78 (1.41–2.33)	0.647
SII	406.87 (303.90–542.58)	405.41 (307.14–543.26)	407.89 (291.34–534.24)	0.546	404.64 (300.03–529.13)	409.64 (316.53–554.29)	0.184
SIRI	0.86 (0.63–1.23)	0.83 (0.61–1.19)	0.93 (0.68–1.33)	0.008	0.86 (0.63–1.23)	0.86 (0.64–1.21)	0.966

BMI, body mass index; LDL-C, low-density lipoprotein cholesterol; ALB, serum albumin; ALT, glutamic pyruvic transaminase; AST, glutamic oxaloacetic transaminase; eGFR, estimated glomerular filtration rate; HGB, hemoglobin.

### 2.3 Disease definition

Sarcopenia was diagnosed according to the 2025 AWGS consensus (Chen et al., 2025), defined as the concurrent presence of low muscle mass and low muscle strength. Appendicular skeletal muscle mass (ASM) was measured using BIA, and the appendicular skeletal muscle mass index (ASMI) was calculated as ASM divided by height squared (kg/m^2^). Low muscle mass was defined as an ASMI <7.6 kg/m^2^ for men or <5.7 kg/m^2^ for women for ages 50–64 years, and <7.0 kg/m^2^ for men or <5.7 kg/m^2^ for women for ages ≥65 years. Muscle strength was measured using a Jamar dynamometer, and the maximum value of three consecutive measurements was recorded. Low strength was defined as a grip strength <34 kg for men or <20 kg for women for ages 50–64 years, and <28 kg for men or <18 kg for women for ages ≥65 years.

### 2.4 Machine learning modeling

Before model development, data completeness and consistency were assessed. Patients with missing key clinical data were excluded according to the prespecified eligibility criteria. Consequently, no missing values were present among the predictors in the final analytic dataset, and no data imputation was performed. The final dataset included 904 eligible participants. Stratified random sampling based on the outcome variable was applied to partition the dataset into a training set (70%, *n* = 633) and a held-out internal validation set (30%, *n* = 271). All feature-selection procedures and model development were performed exclusively within the training set to minimize information leakage. Feature selection was performed using least absolute shrinkage and selection operator (LASSO) regression and the Boruta algorithm exclusively within the training set. The LASSO model was constructed using binary logistic regression, with the optimal penalty coefficient (λ) identified via 10-fold cross-validation based on the one-standard error rule (λ.1se); variables with non-zero coefficients were retained. The Boruta algorithm was implemented with 100 iterations of a random forest model, comparing the importance of original variables against shadow variables to identify features markedly associated with sarcopenia. The intersecting variables selected by both methods were retained as the final predictor set. For tree-based algorithms, no scaling was applied. For SVM, KNN, Neural Network, and Logistic Regression, continuous variables were standardized using Z-scores based on the training set. Variance inflation factors (VIF) were calculated to evaluate multicollinearity.

Eight machine learning algorithms were developed: random forest (RF), gradient boosting machine (GBM), LightGBM, support vector machine (SVM), k-nearest neighbors (KNN), neural network, naive Bayes (NB), and logistic regression. Hyperparameters for all models were optimized *via* 5-fold cross-validation. The discriminative ability of each model was assessed using the area under the receiver operating characteristic curve (AUC), accuracy, sensitivity, specificity, precision, and F1 score, with the optimal probability threshold selected based on the Youden index. Calibration performance was evaluated through calibration plots and the Hosmer–Lemeshow test, while clinical utility was measured by decision curve analysis (DCA). To interpret model predictions, Shapley additive explanation (SHAP) values were calculated to quantify the contribution of each feature.

### 2.5 Statistical analysis

All statistical analyses were conducted using R v.4.3. All continuous variables failed the Shapiro–Wilk normality test and are presented as medians and interquartile ranges (IQRs), whereas categorical variables are described as frequencies and percentages. The Mann–Whitney *U* test was used to compare continuous variables between groups, and the chi-squared test was applied to categorical variables. A two-sided *P*-value of <0.05 was considered statistically significant.

## 3 Results

### 3.1 Comparison of baseline characteristics

A total of 904 patients with T2DM were included in this analysis. The median age of the cohort was 63 (56.75–74) years, and the overall prevalence of sarcopenia was 29.20%. The prevalence of sarcopenia was significantly higher in male patients (33.72%) than in female patients (20.33%) (*P* < 0.05). Participants were randomly assigned to either a training set (70%, *n* = 633) or a internal validation set (30%, *n* = 271), which exhibited sarcopenia prevalence rates of 29.38% and 28.78%, respectively. Except for total cholesterol, baseline parameters were well-balanced and comparable between the two groups.

Significant differences were observed between patients with and without sarcopenia regarding sex, smoking status, alcohol consumption, age, BMI, waist circumference, hip circumference, waist-to-hip ratio, serum albumin, uric acid, urea, hemoglobin, 25-hydroxyvitamin D3, TyG index, METS-IR, TyG-BMI, TG/HDL-C, MHR, and SIRI (*P* < 0.05) (**Table 1**). Univariable logistic regression analysis indicated that 12 variables—including sex, age, BMI, hip circumference, waist circumference, hemoglobin, serum albumin, TyG index, METS-IR, MHR, TG/HDL-C, and TyG-BMI—were found to be significantly associated with the presence of sarcopenia (*P* < 0.05) (**Supplementary Table S2**).

### 3.2 Feature selection

Among the baseline clinical indicators, 9 candidate predictors were identified via LASSO regression, and 15 were selected using the Boruta algorithm. The intersection of these two methods yielded 8 final predictors: age, sex, BMI, serum albumin, alanine aminotransferase (ALT), hemoglobin, METS-IR, and MHR (**Figure 2**). VIF were subsequently calculated to address multicollinearity (**Supplementary Table S3**).

**Figure 2:**
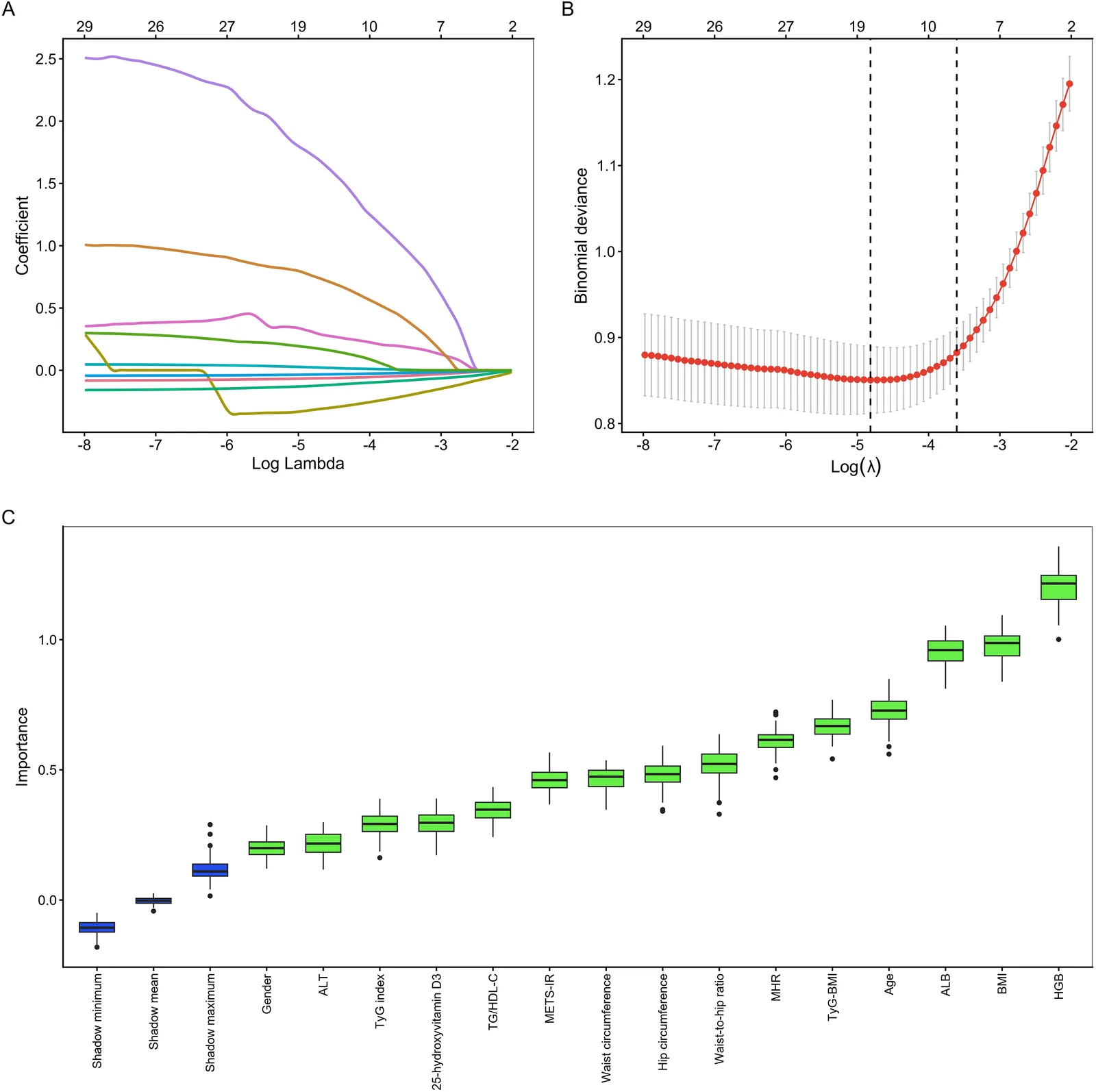
Results of feature selection. **(A)** Variable trajectories in the LASSO model. **(B)** Optimal lambda selection in the LASSO regression with 10-fold cross-validation. **(C)** Boruta feature selection.

### 3.3 Model development and evaluation

Eight machine learning models were developed, and their performance was evaluated across the training set and the internal validation set. In the training set, LightGBM achieved the highest AUC (0.915, 95% CI: 0.892–0.937), followed by GBM (0.906, 95% CI: 0.880–0.929). In the internal validation set, GBM achieved the highest AUC (0.933, 95% CI: 0.899–0.963), followed by LightGBM (0.928, 95% CI: 0.893–0.960) and RF (0.927, 95% CI: 0.893–0.958). The remaining models exhibited lower AUC values, reflecting their poorer discriminative ability.

LightGBM demonstrated the best overall performance among all the models, yielding an accuracy of 0.882, a sensitivity of 0.897, a specificity of 0.876, a precision of 0.745, and an F1 score of 0.814. DCA revealed that LightGBM, GBM, and RF provided high standardized net clinical benefit. Furthermore, calibration curves and the Hosmer–Lemeshow (HL) test indicated that the LightGBM model was well-calibrated (**Table 2**; **Figure 3**). Sensitivity analysis with 50 repeated train-validation splits yielded a mean AUC of 0.893 (SD: 0.0176, 95% CI: 0.859–0.924, range: 0.844–0.926), confirming robust model stability.

**Table 2:** Predictive performance of each model.

Model	AUC (95% CI)	Accuracy	Sensitivity	Specificity	Precision	F1
Training set						
RF	0.899 (0.872–0.923)	0.825	0.79	0.839	0.671	0.726
GBM	0.906 (0.880–0.929)	0.867	0.737	0.922	0.797	0.765
LightGBM	0.915 (0.892–0.937)	0.87	0.753	0.919	0.795	0.773
SVM	0.864 (0.830–0.895)	0.818	0.769	0.839	0.665	0.713
KNN	0.793 (0.768–0.843)	0.682	0.828	0.622	0.477	0.605
Neural Network	0.834 (0.800–0.868)	0.78	0.699	0.814	0.61	0.652
NB	0.757 (0.713–0.798)	0.701	0.704	0.7	0.494	0.581
Logistic	0.854 (0.821–0.884)	0.746	0.849	0.702	0.543	0.662
Validation set						
RF	0.927 (0.893–0.958)	0.852	0.897	0.834	0.686	0.778
GBM	0.933 (0.899–0.963)	0.867	0.872	0.865	0.723	0.791
LightGBM	0.928 (0.893–0.960)	0.882	0.897	0.876	0.745	0.814
SVM	0.890 (0.841–0.931)	0.782	0.923	0.725	0.576	0.709
KNN	0.837 (0.787–0.895)	0.834	0.603	0.927	0.77	0.676
Neural Network	0.842 (0.786–0.891)	0.801	0.705	0.839	0.64	0.671
NB	0.822 (0.766–0.877)	0.756	0.846	0.72	0.55	0.667
Logistic	0.858 (0.802–0.908)	0.764	0.859	0.725	0.558	0.677

**Figure 3:**
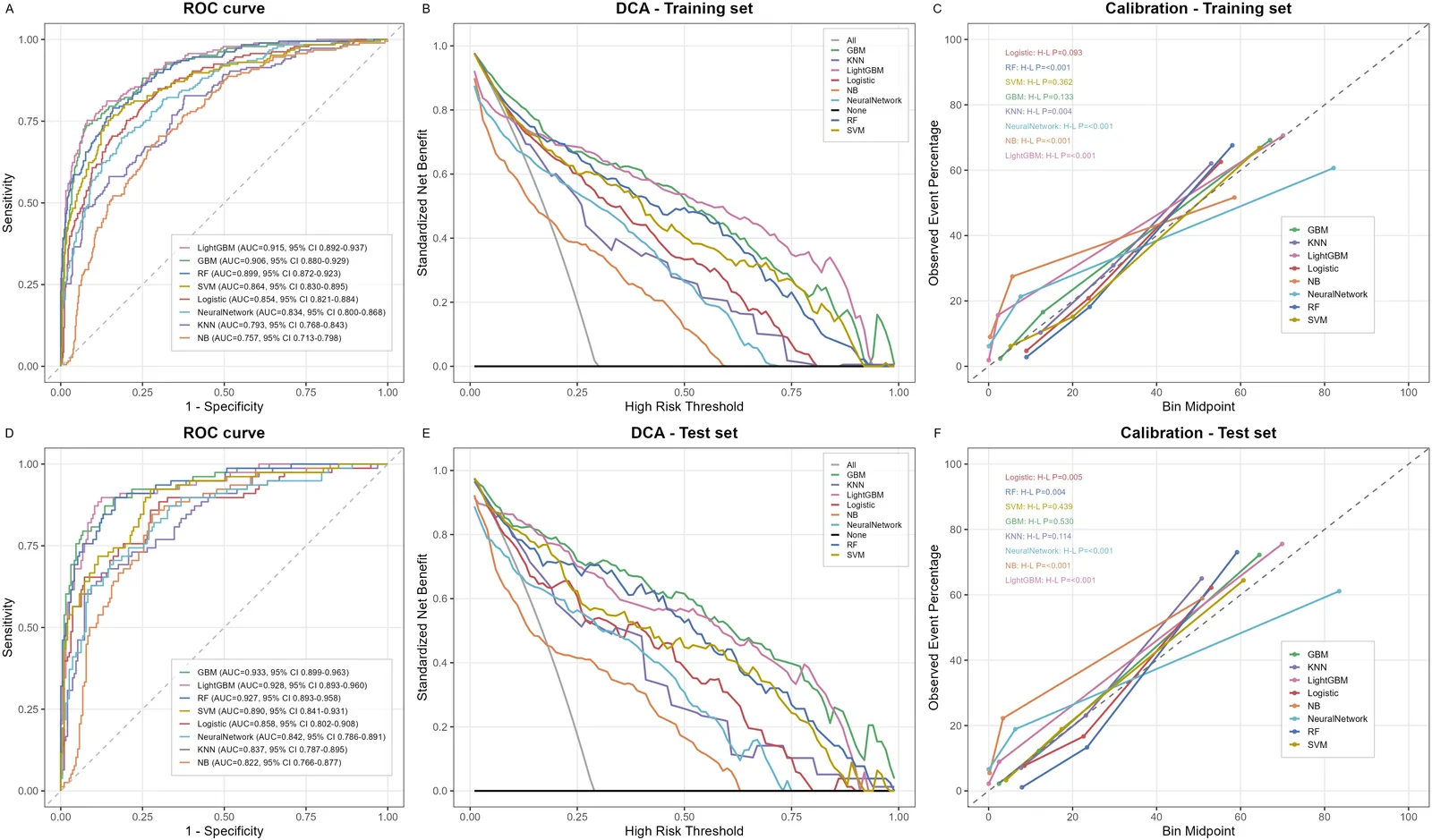
Machine learning prediction models. **(A)** ROC curves for the training set. **(B)** DCA curves for the training set. **(C)** Calibration curves for the training set. **(D)** ROC curves for the validation set. **(E)** DCA curves for the validation set. **(F)** Calibration curves for the validation set.

### 3.4 Interpretability of the optimal LightGBM model

Global feature importance for the eight predictive factors was ranked based on mean absolute SHAP values (**Figure 4A**). As shown in the SHAP summary plot in **Figure 4B**, the relationship between each variable’s values and the corresponding SHAP values was consistent with the global importance ranking. The contribution of each feature to the model’s prediction for a single patient is illustrated in individual SHAP force plots in **Figures 4C, D**. Furthermore, the quantitative association between each predictor and sarcopenia risk is demonstrated via SHAP dependence curves in **Figures 5A–H**.

**Figure 4:**
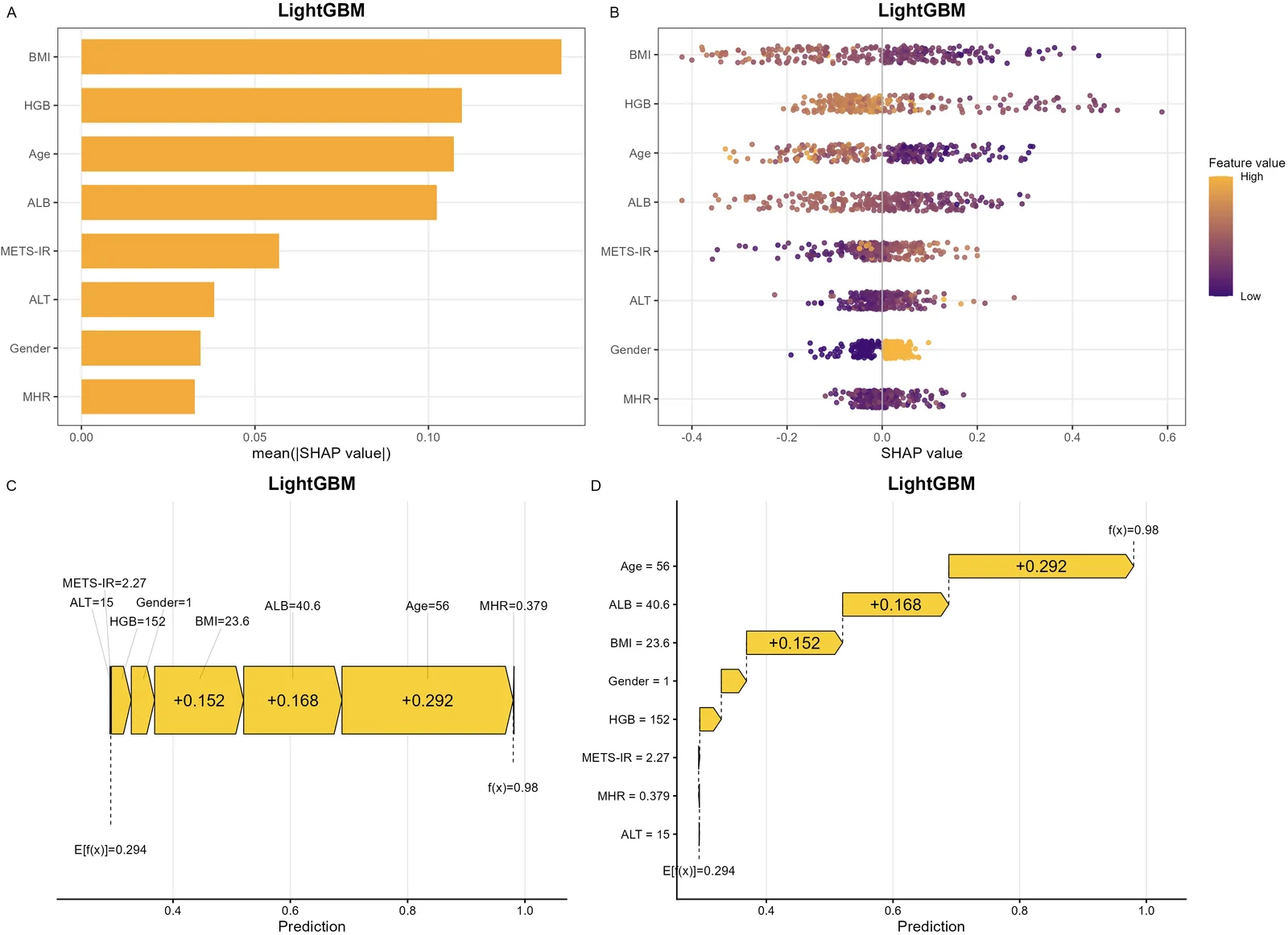
Model interpretability. **(A)** Bar chart of feature importance. **(B)** SHAP summary plot. **(C, D)** Individual SHAP force plots.

**Figure 5:**
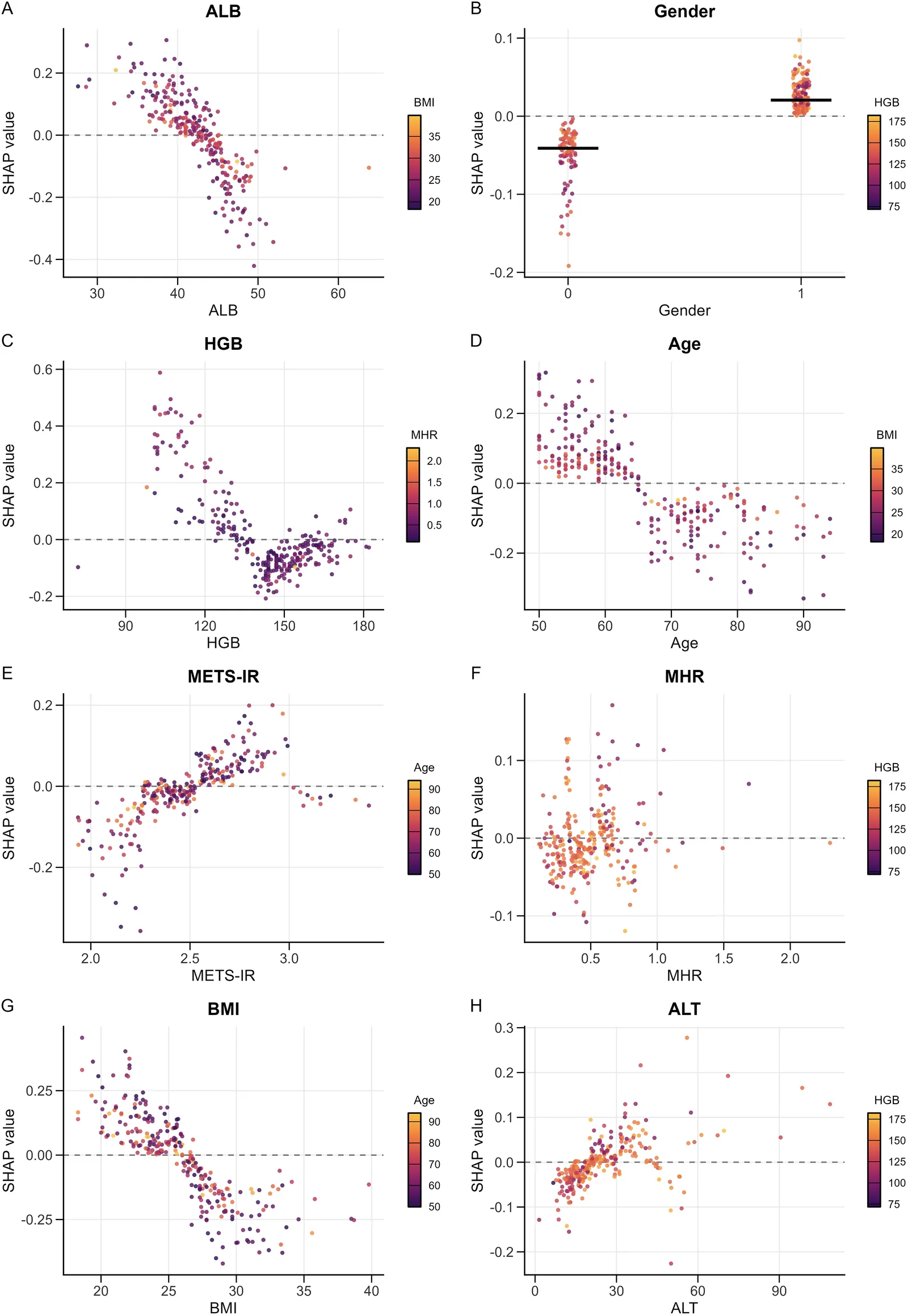
Univariate SHAP dependency plots for **(A–H)** ALB (serum albumin), sex, HGB (hemoglobin), age, METS-IR, MHR, BMI, and ALT (glutamic pyruvic transaminase).

### 3.5 Web-based calculator

Based on these findings, a web-based calculator was developed to provide real-time predictions of sarcopenia risk for patients with diabetes, incorporating a three-level risk stratification: low (<15%), moderate (15%–40%), and high (>40%) (**Figure 6**). This tool is accessible online at: https://sarcopenia-risk.shinyapps.io/sarcopenia-calculator/.

**Figure 6:**
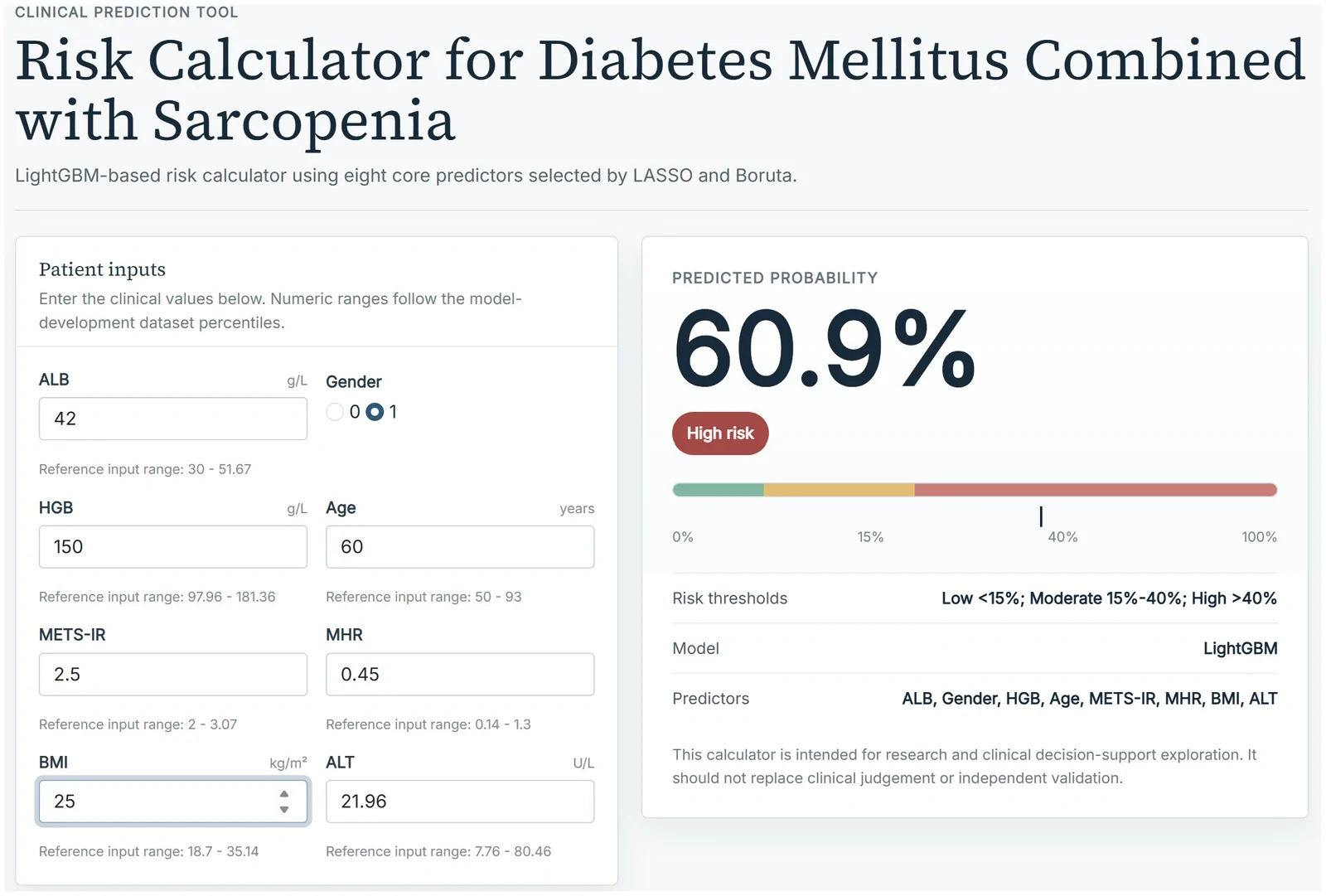
Application of the web-based calculator.

## 4 Discussion

The comorbidity of T2DM and sarcopenia markedly impacts the quality of life of affected older adults and poses a major public health challenge (Wu et al., 2026). These pathologies share a bidirectional relationship, with each potentially exacerbating the progression of the other (Chen et al., 2023; Hou et al., 2023). Consequently, the early identification of high-risk individuals and the timely implementation of clinical interventions are essential. In this study, multiple machine learning prediction models were developed and internally validated based on the AWGS 2025 consensus, incorporating indicators of metabolic-inflammatory crosstalk. Among the eight models evaluated, LightGBM demonstrated superior overall classification performance and was selected for the individualized prediction of sarcopenia risk in patients with T2DM. The resulting model and its online risk calculator provide clinicians with a decision support tool linking mechanistic insights to phenotypic manifestations.

Integrating LASSO regression with the Boruta algorithm yielded eight core predictors, all exhibiting strong clinical applicability and biological plausibility. METS-IR is a reliable surrogate marker for insulin resistance (Bello-Chavolla et al., 2018). Consistent with the findings of Liu et al (Liu et al., 2025b), METS-IR levels were substantial higher in the sarcopenia group than in the non-sarcopenia group. Insulin resistance disrupts the IGF-1/PI3K/AKT/mTOR signaling cascade, thereby suppressing muscle protein synthesis and accelerating proteolysis, ultimately driving muscle atrophy (Giha et al., 2022; Gaur et al., 2024; Ghosh et al., 2026). In this study, the MHR was identified as a key predictive variable reflecting a proinflammatory–anti-inflammatory imbalance (Arabi et al., 2026). These observations indicate that inflammatory–metabolic derangements contribute to the development of sarcopenia (Bektan Kanat et al., 2024; Xue et al., 2025). In T2DM, chronic low-grade inflammation activates the ubiquitin–proteasome system, promoting a decline in muscle mass (Perry et al., 2016).

The prevalence of sarcopenia in T2DM was reported to be considerable higher in male patients than in female patients, a disparity closely linked to sex hormone regulation (Hosoi et al., 2023). While age is an established predictor of diabetic complications (Li et al., 2025; Liu et al., 2025c), it emerged as a protective factor in the current model. This outcome may stem from increased diagnostic rates among individuals aged 50–64 years under the AWGS 2025 consensus, a trend supported by data from Japan (Hashimoto et al., 2026). In our cohort, the prevalence of sarcopenia was 40.1% in the 50–64 age group versus 17.6% in the ≥65 group. This age-prevalence inversion indicates that the observed association is likely a statistical artifact of the updated diagnostic framework rather than a genuine biological protective effect of age. Skeletal muscle mass is maintained through nutritional, metabolic, and tissue-oxygenation mechanisms, which are clinically reflected by BMI alongside albumin and hemoglobin levels (He et al., 2020; Zeng et al., 2022; Zou et al., 2024). Conversely, elevated ALT serves as a risk factor for sarcopenia in patients with T2DM, reflecting an inter-tissue metabolic crosstalk (Okun et al., 2021).

Among the eight machine learning models evaluated, LightGBM demonstrated strong discriminatory ability, satisfactory calibration, and the favorable clinical net benefit, together with the best overall classification performance. These findings corroborate that gradient boosting algorithms are well-suited for predicting sarcopenia risk in patients with diabetes, aligning with a previous large-scale, multicenter investigation (Kara et al., 2025).The internal validation AUC of 0.928 in our model compares favorably with previously reported risk tools for diabetic sarcopenia, which range from 0.800 to 0.932 across various studies (Yin et al., 2025).

All predictors included in the model are routinely available, eliminating the necessity for specialized equipment such as BIA, DXA, or dynamometers. An interactive web-based calculator was developed to facilitate individualized risk estimation and stratification. However, its clinical applicability beyond the current study population requires confirmation through independent external validation. Guided by these risk estimates, clinicians can design tailored interventions, including resistance training and daily protein supplementation, for high-risk populations (Sun et al., 2025). For patients at low to moderate risk, comprehensive follow-up strategies can be customized based on their specific clinical profiles.

Several limitations of this study warrant consideration. First, the single-center retrospective design may introduce selection bias, and the absence of an external validation cohort limits the generalizability of the findings. Second, because the data were collected retrospectively, critical lifestyle and behavioral variables—such as physical activity levels, dietary protein intake, and medication compliance—were absent from electronic medical records and could not be evaluated. Third, although the AWGS 2025 criteria were strictly applied, the study cohort was exclusively Chinese, meaning that the applicability of the model to other ethnic or geographic populations remains to be established. Future multicenter prospective studies using an independent geographic cohort are necessary to externally validate and further refine the proposed model.

In conclusion, in this study, a pathogenesis-based predictive model for sarcopenia in patients with T2DM was developed. The model exhibited favorable discriminative performance with easily obtainable predictive variables, and may serve as a convenient auxiliary tool for early comorbidity screening and individualized clinical intervention.

## Data Availability

The raw data supporting the conclusions of this article will be made available by the authors, without undue reservation.
