# Supplementary material for: Development and validation of a predictive model for sarcopenia in patients with type 2 diabetes based on the synergistic mechanism of insulin resistance and inflammation
Source: Front Physiol. 2026 Sep 21;17:1922889. doi: 10.3389/fphys.2026.1922889 (PMC13634868; doi:10.3389/fphys.2026.1922889)
Supplement: Supplementary file 1 [file Table1.docx]

**Supplementary TABLE S1. Definitions and calculation formulas of the composite indices.**

| **Category** | **Full name** | **Abbreviation** | **Formula** |
| --- | --- | --- | --- |
| Insulin resistance surrogates | Triglyceride-glucose index | TyG | ln[TG (mg/dL) × FPG (mg/dL) / 2] |
|  | Metabolic score for insulin resistance | METS-IR | ln[(2 × FPG (mg/dL) + TG (mg/dL)) × BMI] / ln[HDL‑C(mg/dL)] |
|  | Triglyceride-glucose index combined with body mass index | TyG‑BMI | TyG × BMI |
|  | Triglyceride-to-high-density lipoprotein cholesterol ratio | TG/HDL‑C | TG (mg/dL) / HDL‑C (mg/dL) |
| Inflammatory composite indices | Monocyte-to-high-density lipoprotein cholesterol ratio | MHR | Monocyte count (×10⁹/L) / HDL‑C (mmol/L) |
|  | Neutrophil-to-lymphocyte ratio | NLR | Neutrophil count (×10⁹/L) / Lymphocyte count (×10⁹/L) |
|  | Systemic immune-inflammation index | SII | Platelet count (×10⁹/L) × Neutrophil count (×10⁹/L) / Lymphocyte count (×10⁹/L) |
|  | Systemic inflammatory response index | SIRI | Neutrophil count (×10⁹/L) × Monocyte count (×10⁹/L) / Lymphocyte count (×10⁹/L) |

**Supplementary TABLE S2. Univariate logistic regression analysis for sarcopenia in the training set.**

| **Variable** | **OR** | **Lower 95% CI** | **Upper 95% CI** | **P value** |
| --- | --- | --- | --- | --- |
| Gender | 1.909 | 1.29 | 2.823 | 0.001 |
| Exercise frequency | 1.305 | 0.921 | 1.851 | 0.134 |
| Hypertension | 0.808 | 0.567 | 1.153 | 0.24 |
| Smoking status | 1.394 | 0.963 | 2.019 | 0.079 |
| Alcohol consumption | 1.395 | 0.974 | 1.997 | 0.069 |
| Age (years) | 0.955 | 0.938 | 0.971 | <0.001 |
| BMI (kg/m²) | 0.784 | 0.737 | 0.834 | <0.001 |
| Waist circumference (cm) | 0.976 | 0.96 | 0.992 | 0.003 |
| Hip circumference (cm) | 0.97 | 0.949 | 0.991 | 0.006 |
| Waist-to-hip ratio | 0.185 | 0.017 | 1.982 | 0.163 |
| HbA1c (%) | 1.095 | 0.994 | 1.207 | 0.065 |
| Total cholesterol (mmol/L) | 0.935 | 0.805 | 1.086 | 0.377 |
| LDL-C (mmol/L) | 1.077 | 0.888 | 1.306 | 0.45 |
| ALB (g/L) | 0.848 | 0.809 | 0.888 | <0.001 |
| ALT (U/L) | 1.007 | 0.995 | 1.019 | 0.241 |
| AST (U/L) | 0.995 | 0.977 | 1.014 | 0.6 |
| Uric acid (μmol/L) | 0.998 | 0.996 | 1 | 0.097 |
| Urea (mmol/L) | 1.047 | 0.962 | 1.139 | 0.291 |
| Creatinine (μmol/L) | 1.001 | 0.992 | 1.009 | 0.901 |
| eGFR (mL/min) | 1.006 | 0.994 | 1.017 | 0.334 |
| HGB (g/L) | 0.964 | 0.955 | 0.974 | <0.001 |
| 25-hydroxyvitamin D3 (nmol/L) | 0.997 | 0.988 | 1.006 | 0.458 |
| TyG index | 1.379 | 1.09 | 1.744 | 0.007 |
| METS-IR | 4.255 | 2.047 | 8.846 | <0.001 |
| TyG-BMI | 0.987 | 0.982 | 0.992 | <0.001 |
| TG/HDL | 1.157 | 1.05 | 1.275 | 0.003 |
| MHR | 5.624 | 2.729 | 11.587 | <0.001 |
| SIRI | 1.076 | 0.925 | 1.251 | 0.342 |
| SII | 1 | 1 | 1.001 | 0.836 |
| NLR | 1.008 | 0.908 | 1.12 | 0.876 |

BMI，body mass index; LDL-C, low-density lipoprotein cholesterol;ALB, serum albumin; ALT, glutamic pyruvic transaminase; AST, glutamic oxaloacetic transaminase;, eGFR，estimated glomerular filtration rate; HGB, hemoglobin.

**Supplementary TABLE S3. Variance inflation factor (VIF) estimates.**

| Variable | VIF |
| --- | --- |
| Age (years) | 1.19 |
| Gender | 1.36 |
| BMI (kg/m²) | 1.38 |
| ALB (g/L) | 1.31 |
| ALT (U/L) | 1.18 |
| HGB (g/L) | 1.47 |
| METS-IR | 1.88 |
| MHR | 1.65 |
